# Beyond Workarounds: Enhancing Education, Care, and Wellness on Inpatient Medicine Rotations —A Multicenter Qualitative Study

**DOI:** 10.1007/s11606-025-09392-y

**Published:** 2025-04-15

**Authors:** John Ratelle, Erin Spicer, Kristen A. Bishop, Janet D. Record, Gretchen Colbenson, Aishwarya Kulkarni, Mark Goldszmidt

**Affiliations:** 1 Division of Hospital Internal Medicine, Mayo Clinic, Rochester, MN USA; 2 Division of General Internal Medicine, Department of Medicine, Schulich School of Medicine and Dentistry, Western University, London, Ontario Canada; 3 Centre for Education Research and Innovation (CERI) and Department of Medicine at Schulich School of Medicine and Dentistry, Western University, London, Ontario Canada; 4 Division of Hospital Medicine, Department of Medicine, Johns Hopkins Bayview Medical Center, Baltimore, MD USA; 5 Schulich School of Medicine and Dentistry, Western University, London, Ontario Canada

**Keywords:** medical education, hospital medicine, inpatient teaching, wellbeing

## Abstract

**Background:**

Inpatient medicine rotations (IMRs) aim to deliver exceptional clinical education and high-quality patient care. However, increasing workloads and the fast pace of inpatient wards are undermining this dual objective.

**Objective:**

To explore tensions and challenges between balancing education and clinical practice on IMRs and how physician-leaders are addressing them.

**Design:**

Constructivist grounded theory.

**Participants:**

Inpatient medicine rotation physician-leaders from academic medical centers in the United States and Canada.

**Approach:**

Data collection involved semi-structured individual and group interviews, collected and analyzed iteratively to develop an explanatory conceptual model. Rigor was enhanced through constant comparison, investigator triangulation, and return-of-findings sessions.

**Key Results:**

Twenty interviews involving 27 participants from 20 distinct training programs were conducted. Participants endorsed IMRs unique clinical and educational value. However, they flagged how increasing workloads and resource challenges produce tensions that can undermine the quality of both which, as a consequence, negatively impact attending and trainee wellness. While reactionary “workarounds” were the norm, they often created unanticipated problems. Key IMR features and strategies for success were identified and organized into six categories: (1) patient mix/census; (2) leadership collaboration; (3) collaborative care models; (4) rotation scheduling; (5) clinical workflow; (6) educational workflow. How physician-leaders configured their IMR structures and processes within these categories had the potential to support or undermine the delivery of high-quality care, education, and wellness.

**Conclusions:**

Inpatient medicine rotations, which is essential for clinical care and education, are currently facing serious challenges from a changing clinical and educational landscape. Our findings present a conceptual model highlighting key modifiable variables, giving physician-leaders a framework to assess and enhance their IMR’s clinical learning environment, thus fostering quality care, education, and clinician wellness.

**Supplementary Information:**

The online version contains supplementary material available at 10.1007/s11606-025-09392-y.

## INTRODUCTION

Across the more than 600 Internal Medicine (IM) programs in the United States (US)^1^ and Canada^2^, teams of physicians-in-training care for hospitalized adults under the supervision of IM attending physicians. Known as clinical teaching units (CTU) in Canada^3^ and general internal medicine units in the US^4^, these teams have been a cornerstone of medical education for decades. In this paper, we will refer to them collectively as inpatient medicine rotations (IMRs). Despite their important educational role,^5,6^ IMRs face challenges in balancing clinical workload with high-quality education.^7–9^ Additionally, there is a scarcity of evidence-based practices to navigate these challenges, and limited knowledge about how programs have innovated to overcome them.^10^ Such insights are crucial for program leaders to sustain their IMRs and ensure that high-quality patient care and medical education thrive during challenging times.

Inpatient medicine rotations face numerous challenges, primarily related to increasing workload. These challenges include rising hospital volumes, along with growing patient complexity.^11–13^ This situation is exacerbated by higher patient turnover and a focus on efficiency metrics, with hospitals pressuring IMRs to limit length of stay and discharge patients early in the day to reduce emergency room wait times, despite the lack of robust supporting evidence for benefits of early discharges.^14,15^ Coupled with resident work-hour restrictions^16^ and the demands of the electronic health record and documentation^17,18^, it is difficult for IMRs to allocate time for educational opportunities like didactics and bedside teaching.^7,19,20^ These challenges impact IMRs’ ability to provide excellent clinical care and high-quality education simultaneously, necessitating effective strategies to navigate these obstacles.

While solutions have been proposed to address the challenges on IMRs, they have been variably implemented with mixed effects.^10^ For instance, patient census limits are a necessary mechanism to manage workload on IMRs, but national surveys reveal substantial variation in how and why IMR leaders apply these limits.^20^ This variation may reflect competing considerations among program leaders, such as balancing patient census limits with ensuring residents have ample opportunities to learn by admitting new patients, or a lack of available support mechanisms, such as hospitalist “non-teaching” services, to care for additional patients.^20–22^ The example of patient census limits highlights the complex decision-making and often competing priorities that leaders must navigate when addressing challenges on their IMRs.

Overcoming IMR challenges is not straightforward, and prescriptive solutions alone are insufficient. A deeper understanding of the tensions and challenges experienced on IMRs is crucial. The purpose of this study, therefore, is to explore the tensions and challenges faced on IMRs and the various solutions, both successful and unsuccessful, implemented by physician-leaders. Our goal was to develop a theoretical framework that can effectively guide change efforts to improve both clinical and educational outcomes across IMRs.

## METHODS

### Study Design

We used constructivist grounded theory (CGT) methodology to guide the study design and analysis.^29^ Unlike many qualitative approaches, CGT supports developing theoretically rich explanations of phenomena beyond simple thematic analysis. This rigorous methodology involves several key steps: identifying appropriate participants through purposive sampling; iterative data collection and analysis, using new insights to shape subsequent data collection (theoretical sampling); constant comparison, where older interviews are re-read and re-analyzed as new insights accrue; and iterative coding strategies, starting with open coding, then focused coding, and finally theoretical coding. Data collection terminates once theoretical sufficiency^30^ is achieved.

### Setting and Participants

Participants included IMR physician-leaders such as division chairs, residency program directors, and rotation directors from academic medical centers (AMCs) in the US and Canada. We focused our purposive sampling on AMCs known for educational innovation to uncover strategies addressing the tensions between education and clinical care. In the US, we initially contacted residency program directors at institutions participating in the Educational Innovations Project (EIP).^31^ We supplemented this with a modified snowball sampling method, leveraging the principal investigator’s (JR) professional network and suggestions from EIP leaders to include participants from non-EIP institutions. We emailed 22 institutions in total (11 EIP: 6 university, 5 community programs; 11 non-EIP: 10 university, 1 community program). Individuals from nine US programs agreed to participate (6 EIP, 8 university, 1 community program).

In Canada, we reached out to attending physicians, division chairs, or program directors via a collective email list and the senior investigator’s (MG) professional network, which included representatives from all 17 medical schools. These individuals were invited to participate in interviews or to identify someone knowledgeable about their IMR who could address the research questions. Identified individuals received a standardized recruitment email. Faculty from 13 schools agreed to participate, but due to timing and scheduling conflicts, faculty from 11 schools were interviewed.

### Study Team Reflexivity

The study team comprised clinician educators/researchers with diverse leadership experiences, years in practice, and practice locations, along with an education researcher and a medical student. The team’s diversity, especially in practice locations (Canada vs. US), practice configurations, and years in practice (from a first-year medical student to over 20 years in practice), as well as the inclusion of a PhD non-clinician researcher, enabled us to continually challenge our assumptions and interpretations of the data. Despite this diversity, the team shared a passion for clinical education and a belief in the importance of bedside rounding, which we remained mindful of during interviews and analysis meetings.

### Data Collection and Analysis

The primary data collection method involved semi-structured virtual interviews, lasting between 90 and 120 min, conducted via Western Corporate Zoom. These interviews were audio-recorded and transcribed verbatim using the cloud-based artificial intelligence program NVivo Transcribe. A research team member then de-identified the transcripts. Each interview included at least one participant and two investigators, one from Canada and one from the US. Additionally, three focus groups, each approximately 120 min long, were conducted to refine and clarify findings. Before each interview, participants completed a pre-interview survey about their roles, educational and clinical background, and the structure and processes of their IMR (Supplementary Table 1). Each interview began with a summary and clarification of the survey information to ensure a nuanced understanding of their IMR context.

Consistent with CGT, data collection and analysis proceeded iteratively. The interview guide, initially developed through a literature review, was piloted, refined for clarity, and continuously modified as our understanding evolved. Analysis began with independent, line-by-line coding. This progressed to focused codes applied to several transcripts, eventually leading to a final set of focused codes for all transcripts. Subsequent analysis led to theory development and a final round of coding. Data collection ceased upon reaching theoretical sufficiency around the developed framework.^32,33^

To ensure rigor and trustworthiness of the data, we took several steps: constant comparison and seeking disconfirmatory perspectives during iterative interviewing cycles; investigator memoing and team reflexivity discussions to challenge assumptions and interpretations; and return-of-findings meetings with participants in the final analysis stages to present, clarify, and test our developed theory.

### Ethical Considerations

We obtained ethics approval from Western University Research Ethics Board (REB) (120890) and Mayo Clinic IRB for this Exempt study. This study is reported in accordance with the standards for reporting qualitative research recommendations.^28^

## RESULTS

We conducted 20 semi-structured interviews with 27 participants from 20 training programs (9 US, 11 Canadian). For the return-of-findings focus groups, all participants were invited to participate, and 12 agreed and were available during the scheduled times. Table 1 details participants’ backgrounds and experience.

**Table 1:** Participant Demographics

Characteristic	Value
Country (*n*=27), *n* (%)	
US	9 (33.3%)
Canada	18 (66.7%)
Practice experience (*n*=25*), years, *n* (%)	
1 to 6	2 (8%)
6 to 10	6 (24%)
11 to 15	9 (36%)
16 to 20	1 (4%)
>20	7 (28%)
Experience on current IMR (*n*=25*), year, *n* (%)	
1 to 6	3 (12%)
6 to 10	6 (24%)
11 to 15	9 (36%)
16 to 20	2 (8%)
>20	5 (20%)
Weeks per year attending on the IMR (*n*=24*), *n* (IQR)	12 (3.25)
Completed medical school at IMR center (*n*=27), *n* (%)	6 (24%)
Completed medical school residency at IMR center (*n*=27), *n* (%)	13 (52%)
Current or previous academic roles (*n*=27), *n* (%)	
Site/Medical Director	11 (41%)
Program Director/Associate Program Director	17 (63%)
Clerkship/Course Director	10 (37%)
Division Chair/Chief	11 (41%)
Dean/Associate Dean	2 (7%)
Department Chair/Associate Chair	7 (26%)
Participant gender (*n*=24*), *n* (%)	
Woman	15 (62.5%)
Man	9 (37.5%)
Participant race (*n*=25*), *n* (%)	
White	18 (72%)
Other/mixed	7 (28%)

^*^Number <27 due to items left not answered by participants

*IMR*, inpatient medicine rotation

Despite the contextual differences between the Canadian and US programs (Supplementary Table 2), there were no notable differences in the tensions and challenges of balancing clinical care and education across sites. All participants endorsed the educational value of the IMR and its importance for clinical care and learning.

### Unique Value of the IMR

Interviews consistently highlighted participants’ belief in the unique and valuable educational experience of IMRs within IM training (Supplementary Table 3). Participants noted that while certain skills developed on IMRs are also gained on other rotations, it is on the IMR that trainees “bring all of them together” (Participant 19). This integration is facilitated by exposure to a diverse range of clinical cases and multimorbidity, fostering a comprehensive care approach, encompassing patients’ illness experiences and social determinants of health. Trainees are encouraged to work “shoulder-to-shoulder” (Participant 23) with various kinds of healthcare professionals, progressively taking ownership of their patients. This ownership is supported by an apprenticeship-like model among trainees, with levels of supervision and autonomy varying from students to interns to senior residents, but participants believed that all trainees can “feel like they’re doing the meaningful work” and “contributing to patient care” (Participant 23) at their skill level. Additionally, junior trainees learn by observing their near-peers and setting personal development goals, while senior trainees hone their supervisory, leadership, and teaching skills. Ideally, all this occurs under attending physician’s oversight, promoting ideals of the profession.

### Challenges Facing IMRs

Despite the pivotal role and value of IMRs in medical education, all participants described serious challenges. At the heart of these were the combined issues of high demands, and inadequate resources to meet those demands. In terms of high demands, all participants spoke of increasing patient volumes and complexity (Table 2). As detailed in Table 2, the term ‘complexity’ was not used to describe a singular phenomenon but rather a mix of factors including: an aging population with multimorbidity and the associated struggles of maintaining independence; mental illness and addiction, with limited resources for specialized care; gaps related to the social determinants of health; and complex subspecialty care needs of patients with advanced illness.

**Table 2:** Rising Patient Care Demands on Internal Medicine Inpatient Teaching Services

Demand	Participant quotation
Caring for an aging population	“It’s a big problem in the Canadian healthcare system with our frail, elderly population, we just don’t have enough community supports. And so figuring all of that out actually takes a lot of work.” (Participant 10) “Our cities are getting older and bigger. I see lots more grocery stores and video stores or whatever. I don’t see more hospital beds being made, anywhere, and it doesn’t make sense to me. And that’s not an issue that we can solve here. The aging, the nursing home crisis is real, and I think those real hospital patient issues goes to our residents” (Participant 16)
Mental health and addiction	“Addiction, drug use, mental health access, like whoa. Huge change there. Huge amounts of behavioral issues now in the hospital.” (Participant 1) “The other changing of the demographics is addiction and the government needs to fund more because what can a university do? What can a program director do if they don’t have resources?” (Participant 15) “[Patients] coming in with substance use disorders. Right? We talk about abstinence from these substances and engaging in different activities to get enjoyment or whatever it may be. But then at the end of the day, we’re still struggling with the same problem, the same problem of homelessness exists. And the answer is always not great… Discharge to a homeless shelter, which they’re never going to make it to, and they’re just going to come back 24 hours later in the same alcohol withdrawal picture.” (Participant 21)
Patient complexity and illness severity	“Our house staff are seeing like the most complicated of the most complicated of the most complicated… And even as a generalist inpatient attending, I don’t feel comfortable making some of the decisions on some of the patients.” (Participant 14) “All of a sudden now when every patient is highly complex, has tertiary-quaternary care needs, it’s a different level of complexity. So that is a future that is that’s in my mind, at least is going to be one of the biggest tensions we’re going to have over the next five years.” (Participant 2)
Coordinating care in a complex medical system	“It seems that things have changed in the past like five to 10 years and that these patients are more complex, that their care coordination is more complex.” (Participant 5) “Now it’s like, we’re seeing some really sick patients. With really complex psychosocial situations that are bleeding into what we do, and so and then on layers of complexity of navigating an increasingly complex medical system.” (Participant 1)
Hospital throughput and efficiency	“These type of educational activities that are important for resident growth to make decisions in a safe environment, but it’s not going to be as quick and as efficient as a hospital would like.” (Participant 16) “[IMR trainees] feel the pressure of the system to discharge by 11 and they feel the pressure of flow, which impacts on their ability to participate in educational sessions. That pressure is not coming from us, as attendings, it’s coming from external to the [IMR].” (Participant 9)
Patient volumes	“[We’ve had] problems with how many patients are on a service… too many patients, high variation census.” (Participant 12) “When a team has too many patients, there is no realistic way that they can round on everyone, make decisions, and have appropriate flow happen.” (Participant 16)

*IMR*, inpatient medicine rotation

Compounding the high volumes and complexity of IMR patients were hospital pressures to accommodate growing patient numbers (Table 2). Many participants described hospital leaders and systems focusing almost exclusively on throughput and efficiency, with little appreciation for the “inefficiencies” necessary for quality education. As one participant described:

“Education is just not efficient. Education is redundant. It’s slow… we can get the patient upstairs a lot faster if we don’t involve the trainee. We could get the patient discharged a lot faster if we don’t involve the trainee.” (Participant 2, lamenting the difficulty of maintaining education amid hospital efficiency pressures)

This focus on efficiency is occurring in an “increasingly complex medical system” (Participant 1), where navigating the system to get patients the care they need is becoming more difficult. Consequently, participants described working in the IMR as being fundamentally “harder than it used to be” (Participant 14).

Most participants lamented a related challenge facing IMRs; the fragmentation and eroding quality of multidisciplinary teamwork (Supplementary Table 4). They cited lack of geographic co-location of patients across hospital wards as a key barrier to interprofessional collaboration because effective teamwork “really only happens when you’re geographically situated together” (Participant 11). However, geography was not the sole obstacle to multidisciplinary teamwork. Faced with escalating patient volumes and complexity, both consulting subspecialty physicians and interprofessional healthcare staff are increasingly stretched thin, impairing their ability to forge productive partnerships and a shared understanding of team goals and individual team member roles with the IMR. This often leads to strained working relationships marked by uncertainty over responsibilities and an overreliance on asynchronous communication technologies.

### Wellness on IMRs

Across the interviews, participants consistently emphasized a mounting concern on IMRs: the negative effects of rising IMR challenges on physician wellness. While not explicitly explored in early interviews, wellness concerns and their impact on IMR team member behavior, particularly trainees, were frequently mentioned. Specifically, participants perceived that trainees needed to protect their wellness by spending time away from work:

“The emphasis on getting the resident out, it used to be top down, you know, for better or worse. But now, and not to sound judgmental, I mean, there’s probably some real good to this. But now the residents are like, ‘OK, I’ve got to get out.’ … I think there’s a real tension about not staying longer than you need to.” (Participant 2)

**Figure 1:**
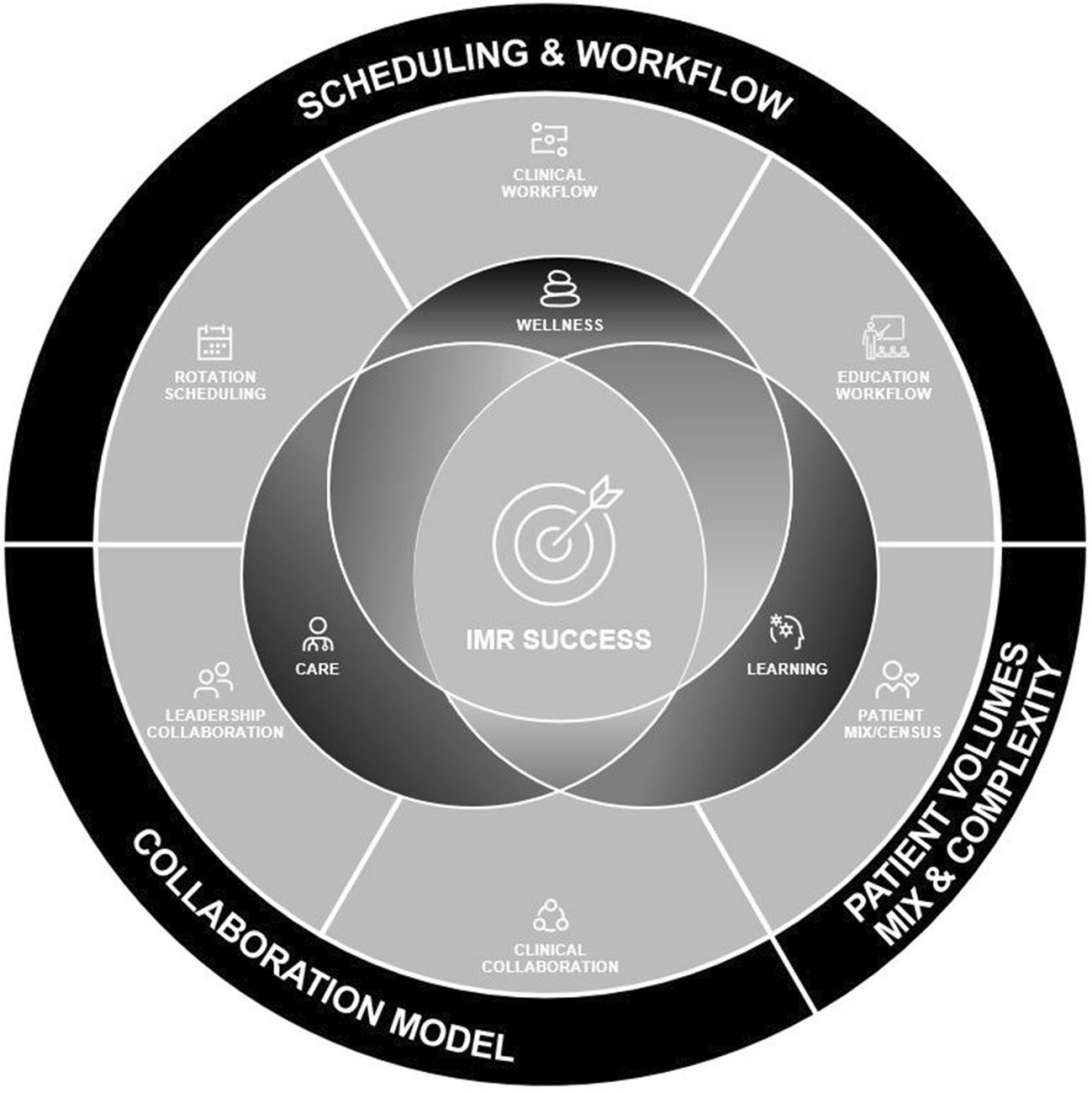
The inpatient medicine rotation (IMR) model: a multi-dimensional framework of the categories of features shaping the IMR experience. A successful IMR designs its features to support patient care, learning, and wellness at work.

As interviews progressed, a theory emerged linking wellness and work, which was tested and confirmed during return-of-findings. Participants felt that wellness can be enhanced both inside and outside work. However, they identified a paradox: a narrow focus on enhancing wellness outside the workplace could lead team members to prioritize leaving work, resulting in a less well workplace. Instead, participants felt that focusing on creating conditions that fostered quality patient care *and* education could naturally enhance workplace wellness (Fig. 1). While participants acknowledged the importance of a reasonable workload, some noted that the most formative experiences occurred on challenging days, leading to “magic” moments where participants perceived that every team member was fully immersed in meaningful work:

“You know, so learner growth, meaningful work, engaging with this woman’s family, and trying to come up with the care plan in the sense that we are doing our best for this woman with a problem that not a lot of other people wanted to treat, including the cardiologists, including the psychiatrists, right? So I felt like that was… a really magic moment.” (Participant 3, describing the experience of caring for a patient with particularly complex needs)

These insights into the relationship between wellness and workplace conditions highlighted the need for a more holistic approach to addressing challenges on IMRs, emphasizing the importance of an environment that fosters high quality patient care and education and allows team members to find meaning in the workplace.

### Reacting to IMR Challenges at the Individual and Program Level: “Survival Mode” and “Workarounds”

Confronted with increasing demands and decreasing wellness on the IMR, participants described predominantly reactive coping strategies at both individual and program levels. These were typically characterized as dysfunctional, largely due to unanticipated ramifications of workarounds (Table 3). While many noted that workplace changes amid the COVID pandemic exacerbated these issues, all participants indicated that this problematic pattern existed before the pandemic.

**Table 3:** Examples of “Workarounds” and Their Unintended Consequences

Challenge	“Workaround” solution(s)	Unintended consequence(s)	Supporting quote
Increasing census on IMRs	Offload low-acuity patients from IMR to non-teaching teams.	Higher acuity among patient admitted to IMR teams.	“what’s happening is because the hospitalists take all the non-acute patients, our [IMR], the patients are becoming more and more sick.” (Participant 19)
	Give the IMR attending physician “private patients” to care for, independent of IMR learners.	Burnout and exhaustion among IMR attendings.	“It’s exhausting, and it’s definitely less good to be an Attending if there’s two private patients you’re separately getting paged on. It’s just like, and then in the morning to show up in the morning. Sometimes you’re post call, you have 15 to staff and you have one or even sometimes two private patients you’ve never met before.” (Participant 7)
High overnight admission volumes	Add extra overnight admitters (trainees or non-teaching attendings).	More handovers and less opportunity for overnight assessment and feedback.	“It has like created that chaos of just like the transitions of care. I think that would be the biggest one is just like things getting like a game of telephone of like, you know, often coming on and being like, well, what’s been done? That hasn’t been done, who’s been called, who hasn’t been called. And then like, you know, not being able to give that feedback sometimes to the to the team from overnight.” (Participant 1)
Work-hour violations on IMRs	Reduce number of admitting days to decrease IMR patient census.	Fewer patients for trainees to learn from.	“It came to the point where our rotation used to be the hardest rotation of residency… then we had residents saying it’s too light and I’m worried about my learning.” (Participant 2)

*IMR*, inpatient medicine rotation

At the individual level, the most problematic coping strategy was described as “survival mode” (Participant 16), where demands on the IMR consistently exceeded available time and resources, prompting team members to seek external means to safeguard their wellness. For attending physicians, safeguarding often meant reducing time spent on their IMR by shortening their rotation duration or serving fewer weeks per year. Whereas faculty previously served two or more weeks at a time, many had reduced their IMR to one week, leading to greater discontinuity of care for patients and less consistent trainee oversight and relationship building.

Trainees, lacking the level of control over their scheduling available to attending physicians, adopted alternative methods to protect themselves. In many centers, especially in Canada, trainees frequently scheduled vacations during their IMR, leading to short-staffed teams and further increasing the demand-resource imbalance. Participants also perceived another shift in trainee behavior was an increased focus on completing the day’s work as quickly as possible to leave the hospital early, sometimes at the expense of their learning:

“Let’s just say expectations have changed pretty dramatically in the last couple of years. I think the residents’ expectation now is that the workday does kind of end at four.” (Participant 8)

In addition to individual coping strategies, participants described how programs are grappling with escalating demands (e.g., increased patient volumes and complexity) on the IMR. To address rising demands and workload, participants mentioned mostly ad hoc “workarounds” implemented without considering the broader context of IMR features. As shown in Table 4, many reactive solutions proved problematic, as “workarounds” often had unintended consequences, leading to new issues: “everything that we’ve tried has backfired in one way or the other. It’s improved some things, and then another unintended consequence has been negative in another arena.” (Participant 30)

**Table 4:** Essential Elements of Internal Medicine Rotation with Supportive or Undermining Features

Domain/category of IMR features	Supportive features	Undermining features
Patient volumes, mix & complexity		
Patient mix/census	• Adjustable census limits based on patient complexity, number of trainees, time of year • Triage officers to support optimal case mix • Non-teaching (hospitalist) teams	• Too many patients • High variation in census • Too many non-acute patients • Too many sub-specialty-focused patients
Collaborative model		
Leadership collaboration	• Leaders’ familiar with IMR needs/priorities • Trainee input on metrics • Hospital metrics adjusted to account for learning	• Antagonistic or indifferent relationship • Lack of resident input • Metrics focused on efficiency and throughput
Clinical collaboration	• Effective teams, collaborative interdependence • Patient geographically co-located • Interprofessional members dedicated to teams • Focus on synchronous communication • Focus on providing care	• Siloed/separated multidisciplinary workflows • Patients geographically dispersed • Interprofessional members shared across teams • Ineffective/asynchronous communication • Focus on discharging patients • 8-5, M-F models of care
Scheduling and workflow		
Rotation scheduling	• Faculty with passion/skill for attending on the IMR • Staggered team changeover • Consistent team size/membership • Adjust trainee rotation duration to match intensity	• Faculty unfamiliar/inexperienced with the IMR • Overlapping changeover days • Inconsistent team size/membership • Trainee rotation duration too long
Clinical workflow	• Mix of independent and supervised time • Adequate time at bedside • Opportunities for interprofessional collaboration	• Divide and conquer to get work done • More direct care by attending • Too little time at the bedside • Prioritizing discharge over care
Education workflow	• Scheduled didactics based on rhythm of day • Range of teaching types, including bedside • Meaningful faculty/trainee engagement • Meaningful observation and feedback	• Scheduled didactics pulling away from learning through care • Lack of faculty/trainee engagement • Lack of direct observation • Heavy focus on accreditation requirements (EPAs, duty-hours)

*IMR*, internal medicine rotation; *EPA*, entrustable professional activity

### The Inpatient Medicine Rotation Model: A Framework to Guide Change Efforts on IMRs

As shown in the Fig. 1 and Table 4, we identified three domains (patient volumes, mix and complexity; collaborative models; and scheduling and workflow) with six categories of modifiable features that influence the IMR experience. While we could not pinpoint a universal set of best practices, participants described general principles for configuring these features to support high quality of care and education on their IMR. Participants also highlighted configurations that can undermine these goals. Notably, the IMR model offers a holistic approach to evaluating a program’s IMR experience, identifying opportunities for improvement and engaging in proactive change.

### Patient Volumes, Mix, and Complexity

To manage rising demands, participants emphasized the importance of patient census limits on IMRs. However, creating static census limits can be challenging and insufficient due to the dynamic nature of workload demands and the team’s ability to meet those demands. Factors such as rotation sequence, turnover, admission timing, case mix, and complexity need to be considered, as well as IMR team size and capability. For instance, new residents or those on their first IMR should manage fewer patients than more experienced residents. Similarly, categorical internal medicine residents might handle more patients than those rotating from other specialties. In Canada, teams can be slightly larger because their third-year medical students function more like US sub-interns and provide patient care with greater autonomy.

Many participants endorsed adjustable census limits that amalgamate patient volumes, workload demands, and team resources and abilities. These limits necessitate creating non-teaching hospitalist teams or increasing subspecialty team admissions to offload extra patients from the IMR. Participants with experience in implementing team caps also highlighted the need for an effective triage process, such as a triage officer, to control and optimize the timing of admission, case mix, and numbers.

### Collaborative Model

Two types of collaboration that directly impact the IMR were identified: first, collaboration with hospital and education leaders, and second, clinical collaboration and the multidisciplinary team.

#### Leadership Collaboration

Participants emphasized the necessity of effective collaboration between clinical leaders (e.g., department chairs, hospital medical directors) and education leaders (e.g., program directors, medical school deans) for the success of their IMR. Particularly valuable were clinical leaders with an educational background who could adjust clinical processes and metrics for educational needs:

“The chief of service was attending [on the IMR] up until he became chief of staff. So we have a particularly good [IMR environment]. There’s no pressure to get someone out by noon, because [he] knows it can’t be done.” (Participant 12)

A few participants highlighted regular meetings between clinical, education, and trainee leaders (such as chief medical residents) as opportunities for productive collaboration. These meetings fostered mutual understanding of each other’s priorities and challenges.

“Once a month, we do quality and outcomes (Q&O). So our vice chair for quality improvement will review our Q&O data with [IMR residents]… the house staff are very bought in to quality improvement and patient safety efforts. And you don’t need to convince them a lot and the Q&O definitely helps with that as well.” (Participant 14)

Conversely, some participants noted that hospital clinical leaders often failed to grasp the education mission, leading to suboptimal collaboration where efficiency metrics took precedence, not out of malicious intent but due to lack of awareness of educational goals.

“Our chief of medicine in terms of the hospital. Again, very nice person. She was a [subspecialty researcher], before she took on this role. And we have annual reviews. And I can’t remember what we talked about during the annual review, but it wasn’t education.” (Participant 15)

Similarly, education leaders disconnected from the IMR clinical environment might prioritize classroom learning (e.g., didactics, academic half days) that clashes with IMR workflow, negatively impacting team members’ ability to participate and learn through patient care. As one participant described; “the academic half day has become a sort of ‘sacred cow’ that is very difficult to change, but it’s totally disruptive for team based work.” (Participant 10)

#### Clinical Collaborative Models

Effective teamwork with other healthcare professionals is fundamental to IMR success and depends on a key latent concept evident throughout the interviews: the ability to form effective relationships among IMR team members and their collaborators. Formulating these relationships is supported by team familiarity, which is best achieved through geographic co-location of patients to single nursing units, because, “[effective teamwork] really only happens when you’re geographically situated together” (Participant 11).

Since geographic co-location was infeasible for many participants’ institutions, other mechanisms to promote team familiarity and effective collaboration included dedicating specific healthcare professionals to collaborate with the IMR. This could include assigning a single case manager, pharmacist, or patient navigator to work with all patients cared for by a single IMR team. This arrangement fostered effective working relationships between these individuals and the trainees on the IMR. Additionally, because these individuals were constant presences in the hospital, they served as connectors to other healthcare professionals the IMR needed to collaborate with. As one participant described:

“We’ve had, for a long time, care navigators who help bridge a lot of [interprofessional communication] and the care navigators stick with the [IMR team] and they’ve been a godsend for us, honestly. So having the care navigator helps that because it doesn’t mean the docs have to do that running around for the complex allied health discussions.” (Participant 10)

Many participants discussed their experiences with asynchronous communication technologies, such as EHR-based messaging systems and pagers, within the domain of clinical collaboration. The general sentiment was that these technologies undermined effective collaboration, but this seemed to depend on how well the IMR team and their collaborators used them. While asynchronous communication technologies had the potential to augment clinical collaboration, their success depended on a foundation of team member familiarity and the shared mental models mentioned earlier. Unfortunately, most participants described a current state where team members who were unfamiliar with each other used asynchronous communication technologies ineffectively, creating strained working relationships and a state of constant distraction:

“It’s incredible. I mean, it’s really, really hard to focus when you’re on some of these [EHR-based text message threads] that you can get 30 messages in 5 minutes and I don’t know how the residents [handle it].” (Participant 7)

### Scheduling and Workflow

Participants described three scheduling elements shaping the IMR experience: one relating to rotation scheduling and two related to IMR workflow.

#### Rotation Scheduling

Table 2 outlines multiple scheduling considerations identified by participants, with trainee-attending continuity as a fundamental priority. At the rotation-level, continuity can be promoted by maximizing the overlap between trainee and attending IMRs, while also staggering changeover among seniors and attendings for clinical continuity as a new team takes over. Equally, if not more important, is the continuity achieved through “consistent team size and membership, because if you don’t have that, when you have less people, [the IMR] has problems dealing with how many patients are on a service.” (Participant 12) Some participants, particularly Canadians, spoke to teamwork and relationship-building difficulties when trainees were gone too frequently while post-call, on vacation, or away at academic half days.

“[The attending] may only see a learner once or twice depending on if they’re post call, the other half day they took a flex day, they went to a conference, etc*.*” (Participant 23)

Other rotation schedule issues include how IMR attendings are selected which, ideally, should be based on their skills as educators and clinicians, as well as familiarity with IMR goals and workflows. Lastly, the optimal rotation duration for trainees also needs to be considered. This was felt to be especially true for senior residents who need a long enough rotation to ensure adequate experience running the team while also recognizing that too long a rotation can lead to burnout due to the intensity of involvement.

#### Clinical and Educational Workflow

Creating an effective workflow that supports both high-quality patient care and education in the current resource-constrained context is a major day-to-day challenge. Importantly, participants discussed the tension not only *between* clinical care and education, such as choosing between early discharges and bedside teaching on rounds, but also *within* care (e.g., balancing efficiency with quality care, like taking time for patient-centered conversations vs. discharging patients early to free up beds) and *within* education (e.g., choosing between attending a noon lecture or staying on the wards to participate in a difficult conversation or perform a procedure).

Ideally, a balance is needed where trainees have sufficient independent time for bedside work, case review, and formulating their own plans, along with opportunities to observe and be observed performing core skills like obtaining consent, breaking bad news, conducting physical exams, and collaborating with multidisciplinary team members. Participants noted that the most valuable learning occurs on the wards, with didactics serving as a supplement. Some programs have adapted their schedules to fit their daily rhythm, such as moving the traditional morning report to a 10:30 am teaching session that all team members can attend. Additionally, some programs developed alternative opportunities for night-float residents, such as weekly post-night sessions to review admissions. Proactive programs collaborated with their multidisciplinary teams to optimize meeting times, ensuring that team members can fully engage in both patient care and education without detracting from other important activities.

## DISCUSSION

Our study, which initially aimed to explore the tensions and challenges between balancing education and patient care on IMRs, found that these tensions were more complex than simply education versus care. This counters our initial assumptions and paints a more nuanced picture. We identified scenarios where care conflicts with care, and education conflicts with education. For example, trainees sometimes face a decision between spending necessary time with a patient at the bedside vs. rushing to discharge them to free up the bed for the next patient. Likewise, educational tensions surface in decisions like choosing between staying on the wards to learn a new procedure versus attending a scheduled didactic session. These insights challenge the prevalent “zero-sum” view of balancing patient care and education on IMRs and replace it with a more nuanced one where complex, contextually sensitive solutions are required.

A surprising insight from our study was the imperative to focus on delivering quality care and education from a wellness perspective. Participants expressed that poorly structured IMRs created scenarios where clinical and educational demands outstripped team members’ time and resources to meet them. This, in turn, promoted dysfunctional strategies focused on self-preservation rather than engagement. This insight is consistent with existing research on workplace burnout and resilience, such as the *job demands-resources model*. This model highlights that when job demands exceed resources (physical, psychosocial, and organizational), some workers (i.e., attendings and trainees) may withdraw from the workplace, which can decrease engagement and worsen collective wellness.^34,35^

Although solutions related to demand-resource imbalance must necessarily be designed in the context of each unique program, IMR leaders trying to address this problem should focus on structural and workflow improvements. For example, participants identified the high volume of secure messaging within electronic health records as a source of stress and frustration. This challenge, also recognized in the broader hospital medicine literature^36^, could be mitigated by enhancing synchronous communication within existing workflows. Bedside interprofessional rounds, for instance, have shown promise in reducing secure messaging volumes and improving satisfaction with communication.^37^

While many of our findings align with existing literature on evidence-based practices for IMRs,^10^ our study underscores the importance of contextualizing solutions within the broader interplay of IMR features. Interventions implemented in isolation or as reactive measures to pressing concerns (e.g., patient volumes) may be less effective. For instance, while bedside interprofessional rounds appear to address asynchronous communication, their success depends on contextual factors such as geographic co-location of patients on a single ward and alignment with nursing workflows. Without these considerations, the efficacy of such interventions may vary substantially, as observed in prior studies.^23,25^

Our findings emphasize the need for a holistic approach to intervention design, considering the interconnectedness of structural, educational, and clinical factors. For IMR leaders, this means not only identifying and addressing ineffective aspects of their teaching teams but also tailoring interventions to the specific demands and resources of their institution. By acknowledging these complexities, leaders can better foster environments that support both patient care and education.

We hope the model derived from our study offers diagnostic insights into undermining features of IMRs and actionable strategies for fostering wellness. As IMR leaders seek to improve the balance of care and education, this model can serve as a guide to designing interventions that enhance learning, patient care, and wellness on IMR teams.

Our study had several limitations. First, it exclusively involved physician-leaders to gain insights from those with extensive knowledge and experience in IMRs’ clinical and educational aspects. However, this approach excluded trainee perspectives, which may differ. Future research should explore the alignment and divergence of findings within this group. Second, the majority of our participants were from university-based academic medical centers, which could limit the transferability of our findings to IMRs in other settings, such as community-based centers with different dynamics and challenges. Lastly, while we refined our conceptual model through multiple feedback sessions with participants, further empirical research is necessary to confirm its effectiveness as a tool for designing the optimal IMR experience, a crucial next step in validating the practical applicability of our theoretical framework.

In conclusion, the IMR is uniquely valuable in medical education, with its success dependent on providing high-quality patient care and learning experiences that foster wellness. However, its effectiveness is challenged by an evolving landscape of increasing patient volumes and complexity, hospital efficiency pressures, and fragmented multidisciplinary care. These demands often exceed team members’ resources and time, forcing sacrifices in patient care and learning. Despite these challenges, IMR physician-leaders recognize its value, believe that positive change is possible, and can look to findings from this study to guide their change efforts and advocacy more proactively.

## Supplementary Information

Below is the link to the electronic supplementary material.

Supplementary file1 (DOCX 30 KB)
