# Supplementary material for: Beyond Workarounds: Enhancing Education, Care, and Wellness on Inpatient Medicine Rotations —A Multicenter Qualitative Study
Source: J Gen Intern Med. 2025 Apr 15;40(9):2063–74. doi: 10.1007/s11606-025-09392-y (PMC12325131; doi:10.1007/s11606-025-09392-y)
Supplement: Supplementary file 1 — Supplementary file1 (DOCX 30 KB) [file 11606_2025_9392_MOESM1_ESM.docx]

**Supplementary Table 1-Pre-Interview Survey Questions**

1. Institution; ______
2. Medical Specialty; _______
3. How many years have you been in practice since the completion of training?
   1. 1-6
   2. 6-10
   3. 11-15
   4. 16-20
   5. >20
4. Other advanced training (E.g., Masters of Education, PhD, Chief Medical Resident, Fellowship, etc.): _______
5. How many years have you practiced at the IMR at your centre since the completion of training?
   1. 1-6
   2. 6-10
   3. 11-15
   4. 16-20
   5. >20
6. How many weeks do you typically work per year on your IMR?: ________
7. Did you do medical school at the same academic centre that you currently work?
   1. Yes
   2. No
   3. Other: __________
8. Did you do residency at the same academic centre that you currently work?
   1. Yes
   2. No
   3. Other: __________
9. Please describe any academic roles that you currently or previously held that might help us to understand your perspectives on the IMR (E.g., assistant program director, quality improvement specialist): __________
10. Please describe any hospital roles that you currently or previously held that might help us to understand your perspectives on the IMR Hospital roles (e.g. IMR site or team leader): How many IMR teams are there at the hospital you work at: __________
11. How many IMR teams are there at the hospital you work at?
    1. 1
    2. 2
    3. 3
    4. 4
    5. 5
    6. >5. Please specify: __________
12. How many rotations on the IMR does each attening do per year? Please describe: __________
13. How do attendings focus their work on the IMR?
    1. Attached to only one IMR
    2. May work across multiple IMR
    3. Other. Please describe: __________
14. What is the composition of learners on the team (I.e. residents, interns, sub-interns, medical students, other). Please specify and explain: __________
15. How are attendings paid? Please check all that apply.
    1. Fee for service
    2. Salary
    3. Hybrid. Please describe: __________
    4. Other. Please describe: __________
16. What percentage of attending physicians are sub-specialists (E.g., cardiologists, respirologists, nephrologists, etc.) on your IMRs? __________
17. In addition to IMR, do you also have a hospitalist service and/or non-teach internal medicine team? Please describe: __________
18. How many attending physicians share supervision at any given time on your IMR?
    1. One attending who is responsible for both teaching and patient care
    2. Two attendings, one teaching attending and one patient care attending
    3. Another configuration. Please describe: __________
19. What is the typical attending physician rotation duration?
    1. 1 Week
    2. 2 Weeks
    3. A mix of 1-2 weeks depending on the attending
    4. Other. Please describe: __________
20. What day of the week do attendings typically take over the team?
21. How many patients does each team (on your IMR) typically take care of?
    1. <10
    2. 10-15
    3. 16-20
    4. 21-25
    5. 26-30
    6. >30
    7. Other relevant patient number details. Please describe: __________
22. What is the typical range of admission numbers/day for each IMR team? Please describe: __________
23. What is the typical range of discharges/day for each IMR team? Please describe: __________
24. How are patients admitted? NOTE: In your description, please include things like who does the admissions and how they are allocated between the different IMR teams and, if relevant to hospitalist and/or non-teach teams for each.
    1. During the day: __________
    2. Overnight: __________
    3. Other comments: __________
25. How many beds are specifically allocated per team?
    1. <10
    2. 10-15
    3. 16-20
    4. 21-25
    5. 26-30
    6. >30
    7. Other relevant patient number details. Please describe: __________
26. Are patients cared for by IMR teams geographically co-located? Please describe: __________
27. How many of each of the following trainee groups work with your IMR at any one time?
    1. Post graduate year (PGY) 5 residents
    2. PGY 4 residents
    3. PGY 3 residents
    4. PGY 2 residents
    5. PGY 1 residents
    6. PGY 1 residents training in a different specialty (E.g., surgical resident rotating through)
    7. 4th year medical students/sub-interns/acting interns
    8. 3rd year medical students/core clerkship students
    9. Other. Please describe any other trainees not captured in list
28. Are there other team members attached to each IMR (e.g., patient navigator, geriatric nurse etc.)? Please specify and describe: __________
29. How is overnight handover and call handled on your IMR? NOTE: In your description, please include things like timing of handover and duration of on-call period etc. Please describe: __________
30. Please describe a typical weekday for your IMR. NOTE:  In your description include things like where rounds take place (bedside vs. Conference room vs. Hallway), the timing and types of rounds/halfdays that pull trainees away from the wards, the timing and types of multidisciplinary rounds that take place etc.): __________
31. Please describe a typical weekend day for your IMR: NOTE: In your description include things like where rounds take place (bedside vs. Conference room vs. Hallway), the timing and types of rounds/halfdays that pull trainees away from the wards, the timing and types of multidisciplinary rounds that take place etc., and any other relevant details: __________
32. Which option(s) best describes your gender identity? Please select all options that apply:
    1. Gender non-conforming, Gender Fluid, Non-binary, Genderqueer, Gender variant, X, Agender, Queer
    2. Man (includes Transman)
    3. Two-Spirit
    4. Trans
    5. Woman (includes Transwoman)
    6. I prefer not to answer
    7. Additional, Please specify
33. Which option(s) best describe the race you self-identify with? Please select all options that apply:
    1. Black (e.g. African ancestry, Afro-Caribbean, African American, African Canadian, etc.)
    2. East Asian (e.g. Chinese, Taiwanese, Japanese, Korean etc.)
    3. Indigenous Global (e.g., Maori, Australian Aboriginal, South American Indigenous, etc.)
    4. Latino/a or Latinx (e.g. South, Central American, Caribbean etc.)
    5. South Asian (e.g. Bangladeshi, Pakistani, Indian, Sri Lankan etc.)
    6. South East Asian (e.g. Vietnamese, Thai, Cambodian, Malaysian, Filipino/a, etc.)
    7. West Asian or Middle Eastern (e.g. Iranian, Afghani, Lebanese, Egyptian, Iraqi, Armenian, Israeli, Palestinian, etc. )
    8. White (e.g. Caucasian, European ancestry etc.)
    9. I prefer not to answer

**Supplementary Table 2-Summary of the dominant differences in Canadian and American Inpatient Medicine Rotation features as described by study participants.**

|  | Canada | United States |
| --- | --- | --- |
| Faculty | Variability around how many weeks/year on IMR. In recent years, the majority had reduced the number of consecutive weeks on call to between 1 and 2 weeks. | Variability around how many weeks/year on IMR. In recent years, the majority had reduced the number of consecutive weeks on call to between 1 and 2 weeks. |
|  | Majority GIM faculty with variable size of attending pool. | Majority GIM faculty from much larger attending pools. |
|  | Minimal adjustment in weeks based on teaching performance. | Weeks are more likely to be based on teaching performance. |
|  | Most were fee for service billing/hybrid. | All were salaried. |
| Capping | Less likely to have capped teams. | All had capped teams. |
|  | If capped, most were in the low 20’s. | Most caps were 15 or less. |
| Non-teaching services* | Present in all programs and could include a mix of family practice and internist run, typically low acuity, teams. | All had some form of non-teaching service run by GIM hospitalists. Many had more non-teaching services than they had IMRs. |
| Daytime Admission model | Most programs had a separate team doing daytime admission, with patients handed over to IMR team daily (i.e., drip model). | All IMR teams did their own admissions, usually as part of a rotating call-cycle (i.e., bolus model). However, number of actual teaching teams/site was similar (2-4). |
| Nighttime Admission Model | Equal numbers of night-float systems vs. IMR on-call systems. | Most programs had separate night-float residents and/or nocturnists to do admissions. |
| Team sizes | Slightly larger teams on average  Occasional Junior Attending physician  1-2 SMRs  2-4 PGY 1  2-4 medical students (with role similar to PGY 1s)  Can also include elective students | Slightly smaller teams on average  Occasional Junior Attending physician  1 SMR  2 PGY 1  2-3 medical students (with less independence and responsibility)  Can include elective students |
| Work Week | Most have a 5 days/week model with weekend on call. | Most have a 7 days/week model with a single day off/week negotiated within the team based on service need. |
| Non-Physician Team Members | Most teams had dedicated non-physician team members. Pharmacists were most common, followed by nurse practitioner/physician assistant. | Most teams had dedicated non-physician team members. Pharmacists were most common, followed by a dedicated social worker/case manager. |

IMR=Inpatient Medicine Rotation

GIM=General Internal Medicine

SMR=Senior Medical Resident

PGY=Post-graduate year

**Supplementary Table 3 Unique Educational Benefits of the Inpatient Medicine Rotation**

| Educational Benefit/Feature | Illustrative Quotation |
| --- | --- |
| 1. Apprenticeship-like learning between students, junior residents, and senior residents | [speaking about IMRs] “it allows for implementation of the apprenticeship model of medical education… my own personal feeling is that's a very important part of medical education. The apprenticeship model where you come on, you’re junior, you don't know what you're doing, but you see the next step up and what they do and vice versa. A lot of training- I mean, the didactic stuff people learn through seminars and that type of thing. But the clinical expertise comes from being a clinical clerk.” (Participant 15) |
| 1. Professional identity formation aligned with the ideals of our profession | “When I think about the learning that occurs between medical students and junior residents and senior residents... it's not just medical factoids and management plans. It's about how to conduct yourself in the hospital*”* (Participant 26) |
| 1. Managing acute illness and chronic multimorbidity | “I personally can't imagine any other way they would see the breadth and acuity that they see on [the IMR]. And like, like I don't know where else they would get that experience and so I think it's important” (Participant 20) |
| 1. Determining what to address in versus out of the hospital and how to support successful transitions of care | “We're going to be thorough. We're going to work things up. All these patient issues… we're going to really work that up and we're also going to really work up this rash that came out of nowhere. And we're also going to optimize their diabetes and we're also going to optimize their hypertension, … that's where the student, that's where the resident, the fellow especially, we want them to start thinking of, ‘okay, yes, the patient is here in hospital. What can we plan for them after hospital?’ We’re not in the outpatient setting currently, but let's plan that follow up. You know, let's plan to optimize their chronic disease. So absolutely, I think we provide the most holistic experience for a patient who's struggling with, you know, illnesses. Right? It's never just one illness.” (Participant 21) |
| 1. Graded autonomy with meaningful participation in patient care commensurate with experience and ability | “I think the [IMR] is very important for our medical students… they feel like they're doing the meaningful work. They feel like ‘I'm contributing to the patient care’, where I remember a lot of rotations where it's like, ‘okay, I am still kind of just the shadow’… so being fully involved, getting a bit of autonomy in talking to your patients and running through plans and getting a sense of agency, I think they get that on [IMR].” (Participant 22) |
| 1. Caring for the whole patient; including psychosocial aspects of illness and health | “In GIM and specifically [IMR], you start to see holistic care, right? So we try to embody that… we're going to be thorough. We're going to work things up... let's plan to optimize their chronic disease. So absolutely, I think we provide the most holistic experience for a patient who's struggling with, you know, illnesses. Right? It's never just one illness.” (Participant 21) |
| 1. Empathy and the need to advocate for patients | “Sometimes advocacy is saying ‘no, I need this right now for my patient’ and sometimes it's not. So they realized, OK, they're still advocating because they're making sure we know what the patient and family would want like, and they're thinking about the goals of care.” (Participant 6, discussing different forms of advocacy on IMRs) |
| 1. Clinical reasoning related to a wide range of clinical topics | “It's not what the attending knows, but how the attending uses his or her information to get to the diagnosis or to get to the right management… attendings challenge them [trainees on the IMR]; ‘Is this really the right diagnosis? Do you really know how to interpret the lab tests and respond to them? Could you be missing anything? What do we have to worry about those kinds of?’ To me, these trump all of the other things because the number one job is to get to the right diagnosis.” (Participant 12, discussing how clinical reasoning is fostered on the IMR) |
| 1. Inter- and intra-professional teamwork skills | “Talking about the uniqueness of [the IMR]. I think it's one of the only opportunities where they [trainees on the IMR] work kind of shoulder-to-shoulder with the occupational therapists and physical therapists. And we do sort of an orientation every block that sort- where they kind of are able to share their scope of practice. And that I think people find invaluable to know what a speech and language pathologist actually does and, and to know what's the difference between occupational therapy and physical therapy and, you know, when to involve the social worker. All of those things are, are pretty key no matter what you end up doing. But knowing people’s scope of practice, I'm not sure that's really taught anywhere else and training.” (Participant 23) |
| 1. Leadership, team management, supervisory and teaching skills (particularly for senior residents) | “The opportunity to run the team... it is a wonderful experience for both sides. The attending physician is able to work with a senior resident. There's mentorship, growth, direct observation” (Participant 17, discussing the value of senior residents’ leading the IMR) |

IMR=Inpatient Medicine Rotation

**Supplementary Table 4 Contributors to Fragmented Multidisciplinary Care on Inpatient Medicine Rotations**

| Demand | Participant quotation |
| --- | --- |
| Geographic “scattering” of patients | “We can do teaching at twenty five, you know, at twenty five, we're able to stay in our home units pretty much with all of our patients. But when it gets bigger than that, we start to scatter. And once you start to scatter it, it just becomes bad for everybody because everybody starts to scatter.” (Participant 10) |
| Interprofessional Staffing Shortages | “right now there's a lot of travel nurses and temporary staff nurses. And that is challenging because they're not quite familiar with the culture and also are much quicker to page for anything.” (Participant 14) |
| IMR Patients Cared for on non-Internal Medicine Units. | “We had like, you know, spine nurses looking after like, like septic shock with like, like respiratory failure and stuff. It wasn't- it was not safe.” (Participant 9)  “It's different on other units; how you build teams that we're not as intimately familiar with. There are cultural differences. There are process differences. I think the main difference is the nursing and nursing expertise on those units.” (Participant 2) |
| Diminishing Subspecialty Support | “Oncology has a very small footprint here, and it's trying to get out of the game completely, which is an ongoing tension. So they have like two beds, so nearly all oncology complications come to us (IMR).” (Participant 10)  “Medicine’s always the dumping ground. There's always going to be conflict between services, everybody's protecting their own turf” (Partcipant 12) |
| Unclear Roles and Responsibilities | “But of course, you know, physicians are pretty self-centered and we always think we work harder than everybody else. And, you know, like, well, why can't a nurse do this? And why can't a physiotherapist uses a, why am I running around doing this? And you know, there's there's some truth to that, right? Like, there are a lot of things that we do as physicians that don't actually require a physician's skill set. OK. But it has to be done. And the question is, who else is going to do it ?” (Participant 4)  “What’s a physician task vs. what the non-physician tasks constantly comes up in the workplace. What is appropriate for the residents. You know, where should that line end?” (Participant 2) |
| Asynchronous Communication Technologies | “The evolution of communication here amongst our interdisciplinary teams. So we use secure chat here on Epic. And that I think I have seen has resulted in residents pretty much being glued to their computers or phones, secure chatting back and forth all day.” (Participant 8) |

IMR=Inpatient Medicine Rotation
